# Book Review

**Published:** 1950-10

**Authors:** 


### Reviews of Books

**The Breast.** Edited by F. D. Saner, M.B., B.Ch., F.R.C.S. Pp. xii, 316. Illustrated. Bristol: John Wright & Sons Ltd. 1950. Price 45s.—A number of distinguished anatomists, pathologists, surgeons and others have contributed to this book, but the result is disappointing. There is very little new and what is old has been better done before. The first three chapters deal with structure and function and their abnormalities. (It is odd to find acute infections of the breast in the chapter on function.) The chapter on examination and diagnosis is confused, a discussion on biopsy technique being interrupted by a description of histological criteria. Mammary dysplasia is dealt with in a barely adequate manner, and it is with some difficulty one extracts the author's views that the relationship to cancer is a very slight one. This section could have been expanded with great advantage and some of the published figures quoted and discussed, instead of the singularly useless pages of selected statistical data at the end of the book. There is a valuable short chapter on second intercostal space biopsy, and the management of breast cancer is fully considered from the surgical and radiological aspects. The final chapter on plastic surgery is one of the best in the book but suffers from compression. Printing and paper are excellent, as are the photo-micrographs. The other illustrations are mostly good, except for the coloured figures which are poor and add nothing to the book. Full-page colour plates of intertrigo and pityriasis versicolour are unnecessary, and presumably account in part for the rather high price of this quite modest volume.

**The Liver.** By KASPER BLOND, M.D., and D. HALER, M.B., D.C.P. Pp. 276. Illustrated. Bristol: John Wright & Sons Ltd. 1950. Price 25s.—In this book, Blond and Haler seek to show that the origin of many diseases can be traced to an inefficient liver. They pursue this theme with spirit. It cannot be said, however, that their reasoning is equal to their invention. Their main contention is the familiar one that inadequacy of the portal circulation may lead to flooding of the body with " toxins " " poisons ", " foreign bodies " and the like, which have not " passed through the liver filter ". The scale upon which this theme is developed has surely never been exceeded. " Bright's disease . . . disseminated sclerosis . . . certain types of the anaemias, rheumatism . . . and anginal attacks . . ." are but a few of the maladies which are ascribed with confidence to the universal corruption arising from this mechanism. None will wish to dispute with the authors their suggestion that the venous system has been insufficiently studied, and that shortcomings in it may be responsible for many disorders in ways as yet unperceived. All the more regrettable is it, therefore, that their attack upon this problem should consist largely of a formidable array of non-sequiturs. Thus, for example, they follow the statement that " angina pectoris is almost unknown in mitral inefficiency " by declaring in the next sentence, as though the matter was thus clarified beyond all misunderstanding: " Therefore in patients suffering from anginal attacks which lead to degeneration we find often a history of infectious diseases such as tonsilitis, rheumatic fever, or influenza ". One wonders in passing how many adults would be able to disclaim the history of tonsillitis or influenza. In fact, controlled series, such as those of Yater and his associates, have failed to establish any connection between coronary affection and antecedent disease. To summarize this study: its conclusions have few merits but those of originality.

**Malignant Disease and its Treatment by Radium:** Vol. 3. By Sir STANFORD CADE, K.B.E., C.B., F.R.C.S., M.R.C.P. Second Edition. Pp. xii, 446. Illustrated. Bristol: John Wright & Sons Ltd. 1950. Price 52s. 6d.—True to its title, this volume deals mainly with malignant disease of breast, thorax, digestive tract, bladder, and female genital organs which can well be treated by radium and X-rays. The author gives an authoritative statement of the best form of treatment by any means, in each stage of the disease. His experience is such that he commands respect from both surgeons and radiotherapists. He is a master of both methods. When surgery is the treatment of choice he recommends it, while if radiation offers the best hope he discusses the treatment in detail. The second edition has been brought up to date in detail, and the author has enlisted the aid of specialists for five of the seven chapters. This volume will be read by all radiotherapists, and will be of great value to all general surgeons and gynaecologists. Pathology and the clinical picture are dealt with at length, as well as treatment. Some space has been devoted to description of X-ray therapy techniques, though not as much as is given to radium. There is an extensive analysis of the results of each form of treatment for each site. The illustrations, index and production are good, while the extensive bibliography is a valuable feature.

**Pye's Surgical Handicraft.** Edited by HAMILTON BAILEY, F.R.C.S. Sixteenth Edition. Pp. xii, 724. Illustrated. Bristol: John Wright & Sons Ltd. 1950. Price 25s.—The sixteenth edition of this work is a still further improvement on previous editions. It is a most valuable reference work, which is likely to prove of real practical value to resident hospital officers, and indeed there is a great deal in it which is of real practical value to general practitioners too. There are several new contributors and a section on emergency dental treatment. There are inevitably one or two minor criticisms which could be made, and among them one might mention that there is no reference to the occurrence of homologous serum jaundice following the use of pooled plasma. The book is a convenient size, the quality of the paper and the illustrations are excellent. It is profusely illustrated by photographs and diagrams.

**Epidemiology in Country Practice.** By W. N. PICKLES. Pp. viii, 110. Illustrated. Bristol: John Wright & Sons Ltd. 1950. Price 10s. 6d. —This book was first published in 1939. In 1941, the type was destroyed by enemy action. In response to numerous appeals it has been republished. Dr. Pickles practices in Wensleydale and gives a lucid and interesting account of his research into the spread of infectious disease in the area: many new facts have been established and the work illustrates the value of research in general practice. This book should be read by every general practitioner.

**Aviation Medicine.** By KENNETH G. BERGIN, M.D., D.P.H., A.F.AE.S. Pp. xiv, 447. Illustrated. Bristol: John Wright & Sons Ltd. Price 35s.—Since the beginning of the last war a great deal of information has been gained concerning the medical problems involved in modern aviation. The author of this book has endeavoured to condense this knowledge into readable and compact form. He has covered practically all the important aspects of the subject and gives a very full bibliography. The physiology in certain sections is not always easy to read and some of the diagrams would be clearer if given fuller and more descriptive legends. It is doubtful if the space given to such subjects as diet and to the effects of alcohol and tobacco is justified in a work of this type. However, the book contains a great deal of valuable information and is plentifully illustrated with good photographs.

**Text-book of Psychiatry.** By Sir DAVID HENDERSON, M.D., F.R.F.P.S., F.R.C.P. and the late R. D. GILLESPIE. Seventh Edition. Pp. xii, 740. London: Oxford University Press (Geoffrey Cumberlege). 1950. Price 32s. 6d.—That this text-book is now appearing in its seventh edition since it was first published in 1927, is a proof of its established place in psychiatric literature. The present edition has been chiefly revised by Henderson, since the tragic death of his collaborator, R. D. Gillespie, in 1945. The general plan of the book differs in no important respect from the sixth edition. The chapter on the relations of psychiatry and law has been re-written to bring it up to date in accordance with recent changes in legislation. In keeping with the style of the author, it is lucid and precise. The chapter on psychopathic states is of special value, as this is a subject of which Sir David Henderson has made a particular study, and it is interesting to note how he differs from some others in attaching no great importance to the relationship between child and mother in the potential psychopath. As before, the book reflects the teachings of the school of Adolf Meyer and emphasizes the importance of activating factors in the production of disease reaction types. While this remains one of the most valuable standard text-books of psychiatry in the English language, more space might have been given to modern developments in physiological and psychological research and less to lengthy case descriptions. Only about eighty out of more than seven hundred pages are devoted to psychoneurotic reaction types; and the bibliography does not on the whole represent adequately the trends of current psychiatric literature.

